# Public health round-up

**DOI:** 10.2471/BLT.20.010520

**Published:** 2020-05-01

**Nurses on the front line**

A nurse takes a girl’s temperature at a primary health care centre in Beirut, Lebanon. Nurses are vital to the functioning of health systems worldwide. According to a new report on the state of the world’s nursing, 6 million more nurses are needed to achieve universal health coverage and the sustainable development goals.

**Figure:**
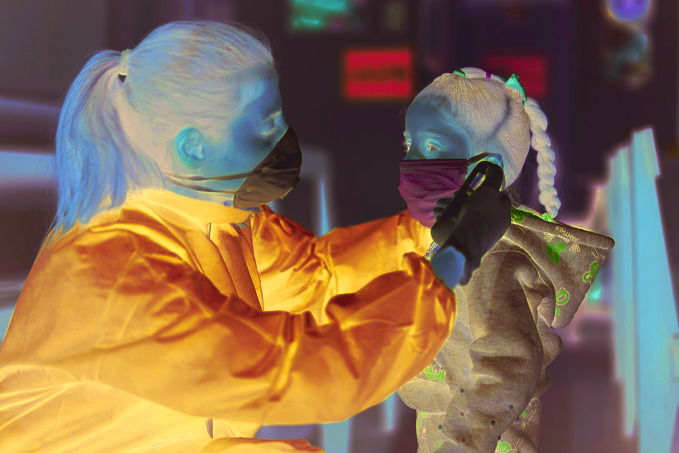
UNICEF/Fouad Choufany

## Measles immunization delayed

An estimated 117 million children in 37 countries may miss out on receiving measles vaccine as a result of delays in campaign implementation. This is according to a statement issued by the Measles & Rubella Initiative (M&RI) on 14 April. M&RI stated that immunization campaigns in 24 countries have already been delayed and more will be postponed.

M&RI noted World Health Organization (WHO) guidance published on 26 March recommending that governments temporarily pause preventive immunization campaigns where there is no active outbreak of a vaccine-preventable disease in order to focus efforts on the COVID-19 response.

WHO also recommends countries continue routine immunization services, while ensuring the safety of communities and health workers.

M&RI stated that its members “strongly agree” with this guidance while urging countries to undertake a careful risk-benefit analysis when deciding whether to delay vaccination campaigns in response to outbreaks.

“The pandemic sweeping the globe requires a coordinated effort and commitment of resources to ensure frontline health workers around the world are protected, as they face and respond to this new threat. At the same time, we must also champion efforts to protect essential immunization services, now and for the future,” the statement said.

M&RI is a global partnership, founded by the American Red Cross, the Centers for Disease Control and Prevention, the United Nations Children’s fund, the United Nations Foundation and WHO.

https://bit.ly/2VoRe1q

## Health systems under strain

WHO released guidelines to help countries maintain essential health services during the COVID-19 pandemic. Published 30 March, the guidelines are designed to help countries meet the demands of mounting a COVID-19 response while maintaining essential health service delivery and mitigating the risk of health system collapse.

The guidelines include a set of actions that countries should consider in order to maintain high-quality essential health services for all.

The guidelines recommend coordinated planning and action to identify essential services to be prioritized to maintain continuity of service delivery.

Previous outbreaks have shown that when health systems are overwhelmed, deaths from vaccine-preventable and treatable conditions can also increase dramatically. During the 2014-2015 Ebola outbreak, for example, deaths caused by measles, malaria, HIV, and tuberculosis attributable to health system failures exceeded deaths from Ebola.

https://bit.ly/2yNcZQO

## More nurses needed

The world needs an estimated 6 million more nurses to achieve universal health coverage and other sustainable development goals.

The estimate is reported in *The state of the world’s nursing 2020*, which was published on 7 April by WHO in partnership with the International Council of Nurses and Nursing Now.

The report reveals that despite a 4.7 million increase in the number of nurses between 2013 and 2018 to just under 28 million nurses worldwide, there is a global shortfall of 5.9 million nurses. 

The report recommends that governments invest in nursing education and the creation of nursing jobs. “This report is a stark reminder of the unique role [nurses] play, and a wakeup call to ensure they get the support they need to keep the world healthy,” said WHO Director General Tedros Adhanom Ghebreyesus.

https://bit.ly/2y4u8Vv

## Lockdown exposing children to violence

Leaders of organisations committed to ending violence against children, called on governments and the international community to unite in protecting children from the risk of violence, exploitation and abuse, which may be heightened as a result of the COVID-19 pandemic response.

School closures have impacted more than 1.5 billion children, while movement restrictions, loss of income, isolation, overcrowding and high levels of stress and anxiety are increasing the likelihood that children experience and observe physical, psychological and sexual abuse at home – particularly those children already living in violent or dysfunctional family situations.

Launched on 8 April, the appeal calls for a collective response including mental health and psychosocial support, social protection for the most vulnerable children, and care and protection for children in institutions.

https://bit.ly/2JWPuaa

## Uniting for COVID-19

WHO and the United Nations Children’s Fund (UNICEF) agreed to work together on the COVID-19 response through the COVID-19 Solidarity Fund.

Announced 3 April, the partnership will see part of the fund allocated to UNICEF which will use the resources to engage communities in the COVID-19 response, as well as to improve access to water, sanitation and hygiene.

UNICEF will also deliver evidence-based guidance delivered through its community outreach and country programs.

“COVID-19 is an unprecedented pandemic requiring extraordinary global solidarity to urgently respond,” said WHO Director General Tedros Adhanom Ghebreyesus. “I’m pleased that UNICEF joined the Solidarity Response Fund. With their extensive experience both in fundraising and in implementing programmes, this partnership will help us to work together closely to save lives.”

The fund will also support the Coalition for Epidemic Preparedness Innovations (CEPI), which is financing research and development for novel vaccines to combat COVID-19 and is working closely with WHO.

The COVID-19 Solidarity Response Fund was launched on 13 March, and is designed as a mechanism for businesses, philanthropies and individuals to contribute to the COVID-19 response effort.

https://bit.ly/2JQQgW7

## New Ebola virus case

A new case of Ebola virus disease was confirmed in the city of Beni in the Democratic Republic of the Congo on 10 April. Prior to this, the last person confirmed to have Ebola in the country tested negative twice and was discharged from a treatment centre on 3 March 2020.

“While not welcome news, this is an event we anticipated. We kept response teams in Beni and other high-risk areas for precisely this reason,” said WHO Director General, Tedros Adhanom Ghebreyesus.

As with all confirmed cases, efforts are already underway to find everyone who may have been in contact with the person in order to offer them the vaccine and monitor their health status.

As of 10 April 2020, 3456 people had been infected with the Ebola virus, 2276 dying as a result.

On 14 April, the International Health Regulations Emergency Committee for Ebola in the Democratic Republic of the Congo advised the Director General that the outbreak continues to constitute a Public Health Emergency of International Concern. The advice was accepted by the Director General.

https://bit.ly/2V1GTcB

**Cover Photo**

Samuel Suárez, 27, a Venezuelan doctor who arrived in Ecuador in 2018, works for the public health system in the remote village of San Francisco, caring for residents and Colombian refugees who fled decades of armed conflict. Since the coronavirus crisis erupted, he has been educating elderly and vulnerable patients on preventive measures.

**Figure:**
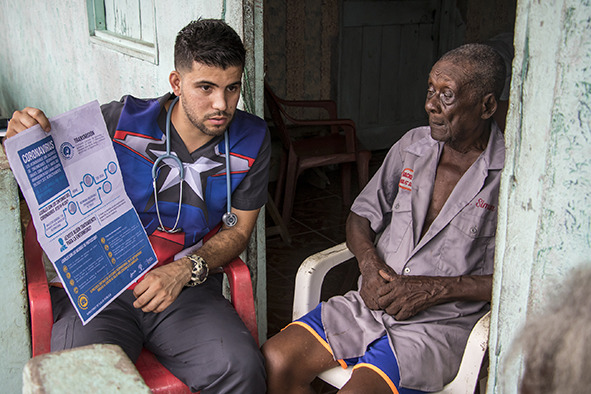
©UNHCR/Jaime Giménez Sánchez de la Blanca

## New guidance on iron status assessment

WHO launched a guideline on the use of ferritin concentrations to measure iron deficiency or risk of iron overload in populations and individuals.

Accurate determination of iron status is crucial for diagnostic and screening purposes in the clinical setting and to guide public health interventions at the population level. In an individual patient, diagnosis of iron deficiency or risk of overload will help guide management, including further investigations and appropriate therapy.

https://bit.ly/2RycOze

## WHO coronavirus timeline

WHO published the timeline of its response to the COVID-19 pandemic in response to media queries. Published on 8 April, the timeline shows that WHO set up an Incident Management Support Team on 1 January, 2020, putting the organization on an emergency footing to deal with the outbreak one day after receiving notification by China of a cluster of cases of pneumonia in Wuhan, China. WHO Director General, Tedros Adhanom Ghebreyesus, declared that the outbreak constituted a Public Health Emergency of International Concern on 30 January.

https://bit.ly/3ekNHd2

## COVID-19 children’s book

An illustrated book aimed at helping children understand COVID-19 was produced by a collaboration of United Nations agencies and nongovernmental organizations that provide mental health and psychosocial support in emergency settings.

Released on 9 April and titled *My hero is you*, the book is aimed primarily at children aged 6-11 years old and is available free online as a text and audio book. It is being translated into more than 40 languages.

“Previous humanitarian emergencies have shown us how vital it is to address the fears and anxiety of young people when life as they know it gets turned upside down,” said WHO Director General, Tedros Adhanom Ghebreyesus. “We hope that this beautifully-illustrated book, which takes children on a journey across time zones and continents, will help them to understand what they can do to stay positive and keep safe during the coronavirus outbreak.”

https://bit.ly/2Rl4yCA

**Looking ahead**

3 – 4 June - Gavi donor conference, London, United Kingdom of Great Britain and Northern Ireland

25 June - Global Summit on Malaria and Neglected Tropical Diseases, Kigali, Rwanda

6 – 10 July - International AIDS Conference, San Francisco and Oakland, United States of America

